# Evaluation of Bluetongue Virus (BTV) Antibodies for the Immunohistochemical Detection of BTV and Other Orbiviruses

**DOI:** 10.3390/microorganisms8081207

**Published:** 2020-08-07

**Authors:** Fabian Z. X. Lean, Jean Payne, Jennifer Harper, Joanne Devlin, David T. Williams, John Bingham

**Affiliations:** 1 CSIRO Australian Centre for Disease Preparedness (ACDP, formerly AAHL), Geelong 3220, Victoria, Australia; Jean.Payne@csiro.au (J.P.); Jenni.Rookes@csiro.au (J.H.); D.Williams@csiro.au (D.T.W.); 2 Department of Veterinary Biosciences, Faculty of Veterinary and Agricultural Sciences, the University of Melbourne, Parkville 3052, Victoria, Australia; devlinj@unimelb.edu.au; 3 Pathology Department, Animal and Plant Health Agency (APHA), New Haw, Addlestone KT15 3NB, UK

**Keywords:** *Bluetongue virus*, immunohistochemistry

## Abstract

The detection of bluetongue virus (BTV) antigens in formalin-fixed tissues has been challenging; therefore, only a limited number of studies on suitable immunohistochemical approaches have been reported. This study details the successful application of antibodies for the immunohistochemical detection of BTV in BSR variant baby hamster kidney cells (BHK-BSR) and infected sheep lungs that were formalin-fixed and paraffin-embedded (FFPE). BTV reactive antibodies raised against non-structural (NS) proteins 1, 2, and 3/3a and viral structural protein 7 (VP7) were first evaluated on FFPE BTV-infected cell pellets for their ability to detect BTV serotype 1 (BTV-1). Antibodies that were successful in immunolabelling BTV-1 infected cell pellets were further tested, using similar methods, to determine their broader immunoreactivity against a diverse range of BTV and other orbiviruses. Antibodies specific for NS1, NS2, and NS3/3a were able to detect all BTV isolates tested, and the VP7 antibody cross-reacted with all BTV isolates, except BTV-15. The NS1 antibodies were BTV serogroup-specific, while the NS2, NS3/3a, and VP7 antibodies demonstrated immunologic cross-reactivity to related orbiviruses. These antibodies also detected viral antigens in BTV-3 infected sheep lung. This study demonstrates the utility of FFPE-infected cell pellets for the development and validation of BTV immunohistochemistry.

## 1. Introduction

Bluetongue virus (BTV) belongs to the genus *Orbivirus* within the family *Reoviridae* and is transmitted by *Culicoides spp.* of hematophagous midges. Various ruminant species can be infected by BTV, including cattle, sheep, deer, goats, and wild ruminants [1,2]. The clinical outcome of BTV infection is dependent on the virus strain, host species, and breed. Infection can range from subclinical to a severe fatal disease, with multiple organ haemorrhage as a result of vascular injury and cytokine release [3].

The BTV genome consists of 10 segments of double-stranded RNA encoding 7 structural and 5 non-structural (NS) proteins [4,5]. The genomic elements are enclosed within a bi-shelled core particle made up of an inner VP3 layer and an outer layer comprised of VP7. The core structure is overlaid with a more diffuse outer coat comprising proteins VP2 and VP5 [5]. The antigenic variability within VP2, which encodes the majority of neutralizing epitopes, determines the distinct serotypes of BTV [6]. Twenty-six BTV serotypes are currently recognised by the *Office International des Epizooties* (OIE) [7]. Recent reports also indicated the presence of other novel serotypes such as 27 and 28 [8,9,10,11,12] and a putative serotype 29 [13]. Among orbiviruses, the antigenic variation within VP7 delineates BTV from other related orbiviruses, such as epizootic haemorrhagic disease virus (EHDV) and African horse sickness virus (AHSV) [14].

During BTV replication, five non-structural proteins (NS1–5) are synthesized. Non-structural protein 1 generates virus-specific tubules in the cytosol during BTV replication and is involved in enhancing virus mRNA translation [15]. The NS2 protein binds individual BTV mRNA, protects transcripts from ribonuclease cleavage, and regulates genome trafficking and packaging; the assembly of NS2 monomers also results in the formation of viral inclusion bodies [16]. Subsequent viral egress in infected insect cells is facilitated by the glycoprotein NS3/NS3a via the calpactin-dependent exocytic pathway [17]. Unlike the other non-structural proteins that are cytoplasmic, a newly identified NS4 protein, encoded by a frameshift open reading frame (ORF) on segment 9, is nucleolar and may be involved in disruption of the host interferon response during viral infection [18]. The functional importance of a putative second ORF within segment 10 remains to be elucidated [4]. Nevertheless, numerous studies have reported NS proteins as being highly conserved [19,20], thus NS proteins could be suitable targets for antigen detection using BTV-specific antibodies.

Currently, there is limited information on characterized BTV antibodies that could enable the detection of viral proteins within infected animal tissues by immunohistochemistry (IHC) [21]. This in turn limits the diagnostic and research applications of animal tissues that might otherwise be possible. During early BTV research, immunohistologic detection of BTV infection in animal tissues relied on immunofluorescence detection on frozen tissue sections and the use of a cocktail of antibodies [22]. However, these immunolabelling techniques were not satisfactory and often not reproducible [22]. The use of formalin fixation on tissues has also presented challenges for the detection of BTV antigens on formalin-fixed paraffin-embedded (FFPE) specimens as formaldehyde can cause cross-linkage of immunogenic epitopes [22,23]. Recent work has shown cross-linking of antigens by formalin can be partially overcome by heat-mediated antigen retrieval [24,25,26]. In these studies, IHC has been successfully applied for the detection of a limited number of strains of BTV on infected animal tissues, including the Netherlands BTV-8 and Italian BTV-1 [24,26], as well as our recent study using the Cyprus BTV-3 strain [25]. Further investigation of the immunoreactivity of BTV antibodies on FFPE material has yet to be performed to determine their suitability for the detection of a broader range of BTV and other orbviruses.

In this study, BTV antibodies were evaluated for their potential to detect viral proteins NS1, NS2, NS3/3a, VP2, and VP7 using heat mediated antigen retrieval IHC. The antibodies were screened for immuno-reactivity against BTV-1-infected FFPE cell pellets (a substitute for BTV infected tissues) followed by evaluation for cross-reactivity to a diverse range of BTV isolates and other orbiviruses. To confirm that the antibodies also detect BTV in animal tissues, we tested them in BTV-3 infected sheep lung. We demonstrated that BTV antigens derived from FFPE BTV infected cells or tissues can be reliably detected by a chromogen substrate-based immunolabeling method.

## 2. Materials and Methods 

### 2.1. Cell Lines and Viruses

A variant of baby hamster kidney cells (BHK-BSR) was grown in basal medium Eagle (BME) (Invitrogen, Australia) and supplemented with 5% v/v fetal bovine serum (FBS) (Invitrogen), 2 mM glutamine (Invitrogen), 100 IU/mL of penicillin, and 50 μg/mL of streptomycin (Sigma, St. Louis, MO, USA). Cells were grown at 37 °C in a humidified cabinet supplemented with 5% CO_2_.

BTV and other orbiviruses were propagated in BHK-BSR cells and BME supplemented with 2.5% v/v FBS, 100 IU/mL of penicillin, and 50 μg/mL of streptomycin at 37 °C. Virus stocks were retrieved from the −80 °C virus repository at CSIRO Australian Centre for Disease Preparedness (ACDP). The viruses used in this study included representative BTV reference serotypes 1 to 24, originally obtained from the Onderstepoort Laboratory, Republic of South Africa (BTV-1 to -8, -10 to -16, -18, -19, -22, and -24), Australian prototype BTV isolates (BTV-1 to -3, -5, -7, -9, -12, -15, -16, -20, -21, and -23), and isolates from the USA (BTV-2, -4, -11, and -17; United States Department of Agriculture, Wyoming) and the Netherlands (BTV-8; Wageningen University & Research, Lelystad). African horse sickness virus (AHSV serotypes 1 and 2) was also obtained from the Onderstepoort Laboratory. Other orbivirus isolates tested included epizootic haemorrhagic disease virus serotype 1 (EHDV-1), Wallal virus, Warrego virus, Eubenangee virus, Yunnan orbivirus-2 (Middle Point orbivirus), Peruvian horse sickness virus (PHSV; Elsey virus), and Palyam virus (Research Centre for Veterinary Science, Indonesia). Full details of virus isolates used can be found in Table S1.

### 2.2. Preparation of BTV and Other Orbivirus-Infected Cells

Culture flasks (75 cm^2^) with 90% confluent monolayers of BHK-BSR cells were infected at a multiplicity of infection of 0.01 with a single isolate of BTV or another orbivirus. Upon the development of 60 to 90% cytopathic effect, cells were fixed with 10% neutral buffered formalin for 24 h to mimic the duration of tissue specimen fixation. Fixed cells were scraped from the flask and along with detached infected cells centrifuged at 1500× *g* for 5 min to sediment the cell pellet. The cells were then re-suspended in a small volume of clear nutrient agar (2.5% w/v nutrient broth and 1% w/v bacteriological agar in distilled water; Media Preparation Unit, University of Melbourne), which was allowed to set before processing for histology. Cell pellets were prepared with single-use instruments and allocated into individual histology cassettes to prevent cross-contamination.

### 2.3. Infected Sheep Tissue

Antigen detection was confirmed on infected lung tissue obtained from an experimentally infected sheep [27]. The lung tissue was obtained from a sheep that was humanely killed seven days after inoculation with a BTV-3 Cyprus 1943 strain of virus. The study was conducted with approval from the ACDP Animal Ethics Committee (#1876), and in accordance with the NHMRC Australian Code for the Care and Use of Animals for Scientific Purposes [28]. The lung tissue was confirmed infected with BTV by detection of segment 10 nucleic acid by reverse transcriptase quantitative polymerase chain reaction, as previously described [29].

### 2.4. Antibodies

The majority of BTV antibody reagents used in this study were either previously purified monoclonal antibodies derived from mouse ascites fluid preparations or derived from cell cultures grown in FBS-free media and used as cell culture derived supernatants at AAHL [30]. The list of antibodies tested in this study is shown in Table 1. A BTV-1 VP7 protein expressed in *Spodoptera frugiperda* 21 was used to produce specific rabbit antiserum at AAHL using a previously described protocol [31]. A rabbit polyclonal antibody (pAb) to the BTV-10 NS2 protein was obtained from Professor Massimo Palmarini (University of Glasgow) [24]. Monoclonal antibodies (mAb) 465 and 441, raised against BTV-10 NS2 and NS3/3a, were obtained from Professor Polly Roy (The London School of Hygiene & Tropical Medicine) [32].

### 2.5. Immunohistochemistry

FFPE cell pellets (suspended in agar as described above) were processed by standard histological methods. Immunohistochemistry was performed on BTV- or other orbivirus-infected BHK-BSR FFPE cell pellets or FFPE sheep tissue that was confirmed with BTV infection. Paraffin wax embedded specimens sectioned at 4 μm thickness were collected on microscope slides (Dako Envision Flex; Agilent Technologies, Mulgrave, VIC, Australia). Sections were then dewaxed through xylene and rehydrated with absolute alcohol, followed by 70% alcohol and water. To retrieve antigens, the sections were heated at 97 °C for 20 min in a bath (water PT Link; Agilent Technologies, Mulgrave, VIC, Australia) containing pH 9 buffer (EnVision™ FLEX target retrieval solution; Agilent Technologies, Mulgrave, Australia). Sections were then rinsed in Tris wash buffer (0.05 mol/L Tris/HCl, 0.15 mol/L NaCl, 0.05% Tween20, pH 7.5) (Agilent Technologies, Mulgrave, VIC, Australia) and treated with 10% peroxidase-blocking agent (EnVision™ FLEX Peroxidase; Agilent Technologies, Mulgrave, VIC, Australia) for 10 min. The primary antibodies were diluted in Envision antibody diluent (Agilent Technologies, Mulgrave, VIC; Australia) and incubated for 60 min at room temperature (RT). For the purpose of screening immunoreactivity against BTV-1 infected cells, antibodies were used at 1:100 without a secondary polymer. For subsequent cross immunoreactivity tests, the working dilutions of the antibodies, as determined from serial dilution testing, were used as follows: NS1 mAbs 31D11B10 and 20A810 (1:1000), NS2 mAb 30G4B10 (1:6000), NS2 mAb 465 (1:2000), NS2 pAb 53414-1 (1:4000), NS3/3a mAb (1:800), VP7 pAb 20-3 (1:1000). Sections labelled with mAb were further incubated with a secondary polymer (Envision Flex mouse linker; Agilent Technologies, Mulgrave, VIC, Australia) for 15 min at RT. Subsequently, slides were incubated with horse-radish peroxidase (HRP)-conjugated secondary antibody (anti-rabbit and anti-mouse) (Envision Flex/HRP; Agilent Technologies, Mulgrave, VIC, Australia) for 20 min at RT. The slides were washed with Tris buffer between each incubation step. Finally, 3-amino-9-ethylcarbazole (AEC) substrate (Agilent Technologies, Mulgrave, VIC, Australia) was added onto the slides for 10 min, which were subsequently counter-stained with Lillie Mayer’s haematoxylin (Australian Biostain, Traralgon, VIC, Australia) and cover slips applied using aqueous mounting medium (Agilent Technologies, Mulgrave, VIC, Australia). Negative control sections were derived from mock-infected BHK-BSR cells.

### 2.6. Immunoblotting

BHK-BSR cells infected with BTV-1 CSIRO 156 were lysed by freezing at −80°C for 24 h and thawing. Cell culture medium (BME), treated in the same manner, was used as a negative control. Lysates were centrifuged at 85,000× *g* for 1.5 h at 4 °C. Pelleted materials were dissolved in 200 µL Laemmli sample buffer, 50 mM dithiothreitol (DTT) (Sigma-Aldrich, Castle Hill, NSW, Australia), and heated at 100 °C for 5 min. Samples diluted in DTT containing Laemmli sample buffer were resolved by sodium dodecyl sulphate–polyacrylamide gel electrophoresis (SDS-PAGE) at 200 V for 40 min using a 4–15% Mini-PROTEAN TGX gel in a Mini-PROTEAN Tetra Cell (Bio-Rad, Gladesville, NSW, Australia). Separated proteins were electro-transferred onto polyvinylidene difluoride (PVDF) membrane (Bio-Rad) using a Trans-Blot Turbo Transfer System (Bio-Rad, Gladesville, NSW, Australia). Membranes were blocked by incubating in 10 mL TBST (20 mM Tris pH 7.4, 150 mM NaCl (TBS), 0.05% (v/v) Tween-20) containing 5% (w/v) skim milk, and then probed with the following primary antibodies: NS1 mAb 31D11B10 and 20A810 (1:1000), NS2 mAb 465 (1:1000), NS2 mAb 30G4B10 (1:2000), NS3/3a mAb 441 (1:1000), VP7 polyclonal antibody 20–3 (1:5000). Subsequently, the membranes were incubated with HRP-conjugated sheep anti-mouse (Sigma) or goat anti-rabbit (Bio-Rad) secondary antibodies (1:2000). Blocking and antibody incubations steps were performed for 1 h each at RT on a rocking platform mixer. Membranes were washed three times for 10 min per wash in 50 mL TBST between incubations. Chemiluminescence was developed with Clarity Western ECL substrate (Bio-Rad) and membranes were photographed using a ChemiDoc Touch Imaging System (Bio-Rad, Gladesville, NSW, Australia).

## 3. Results

### 3.1. Screening and Characterization of BTV-Specific Antibodies

**Figure 1:**
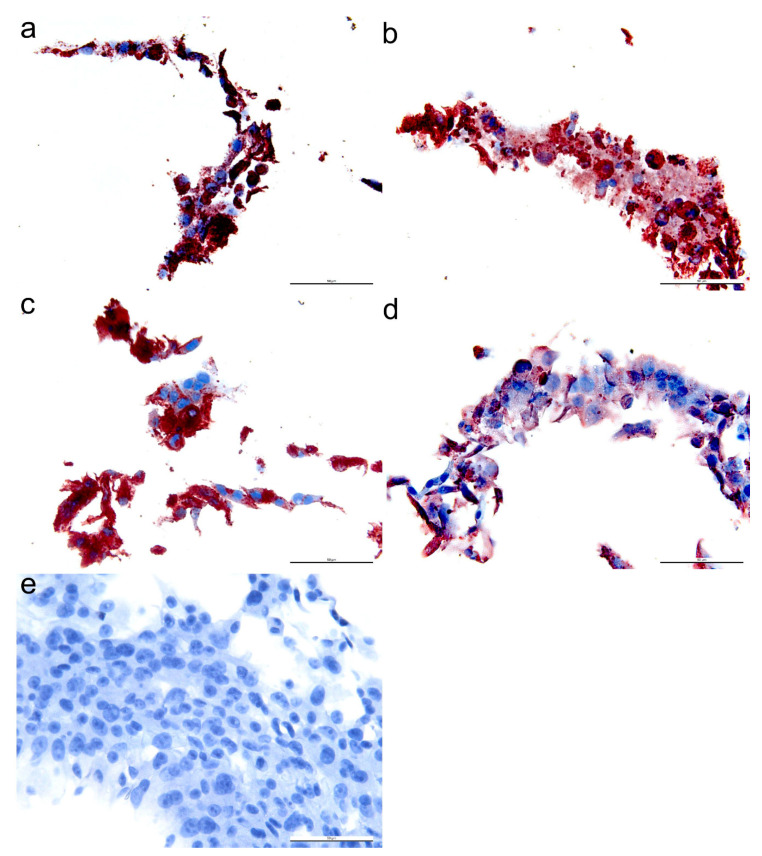
Immunohistochemical labelling of bluetongue virus (BTV)-1-infected baby hamster kidney cells (BHK-BSR) using BTV-specific antibodies. Non-structural (NS) 1 monoclonal antibody (mAb) 31D11 (**a**), NS2 mAb 30G4 (**b**), NS3/3a mAb 441 (**c**), and VP7 polyclonal antibody 20-3 (**d**). A representative image of immunolabelling of uninfected BHK-BSR cells with antibody NS2 mAb 30G4 (**e**). Bar = 50 μm.

**Figure 2:**
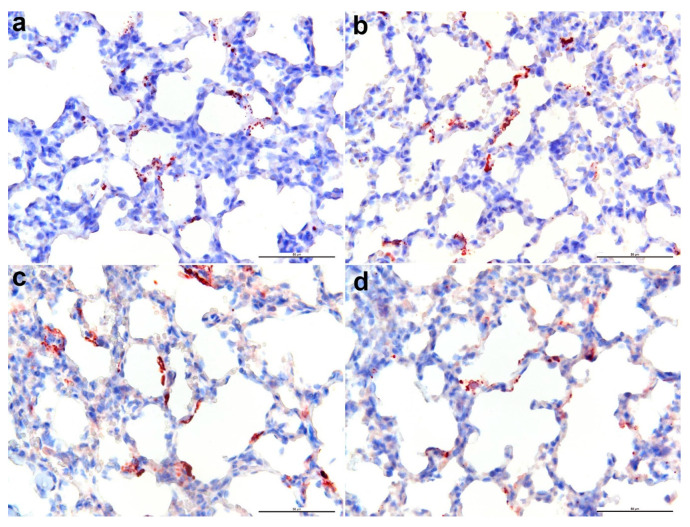
Immunohistochemical labelling of BTV infected sheep lung with BTV-specific antibodies. NS1 monoclonal antibody (mAb) 31D11 (**a**), NS2 mAb 30G4 (**b**), NS3/3a mAb 441 (**c**), and VP7 polyclonal antibody 20-3 (**d**). Bar = 50 μm.

Antibodies raised against BTV structural and non-structural proteins were screened by IHC detection of fixed and embedded BHK-BSR cells infected with BTV-1 CSIRO156 (Table 1). The antibodies that demonstrated specific cytoplasmic labelling against BTV-1 CSIRO 156 was as follow: 31D11 (Figure 1a; NS1 mAb), 20A8 (NS1 mAb), 30G4 (Figure 1b; NS2 mAb), 465 (NS2 mAb), 53414-1 (NS2 pAb), 441 (Figure 1c; NS3 mAb), and 20-3 (Figure 1d; VP7 pAb). These antibodies did not immunolabel uninfected cells (Figure 1e). Several VP2-specific mAbs were tested by IHC, but no immunoreaction was observed. The details of other BTV-specific antibodies that did not produce positive immunolabelling against BTV-1 antigens in infected BHK-BSR cells are shown in Table 1. To determine the utility of the antibodies on infected animal tissues, IHC was performed on BTV infected sheep lung. Specific immunolabelling was detected with BTV antibodies against NS1 (Figure 2a; 31D11 mAb), NS2 (Figure 2b; 30G4 mAb), NS3/3a (Figure 2c; 441 mAb), and VP7 (Figure 2d; 20-3 pAb).

**Figure 3:**
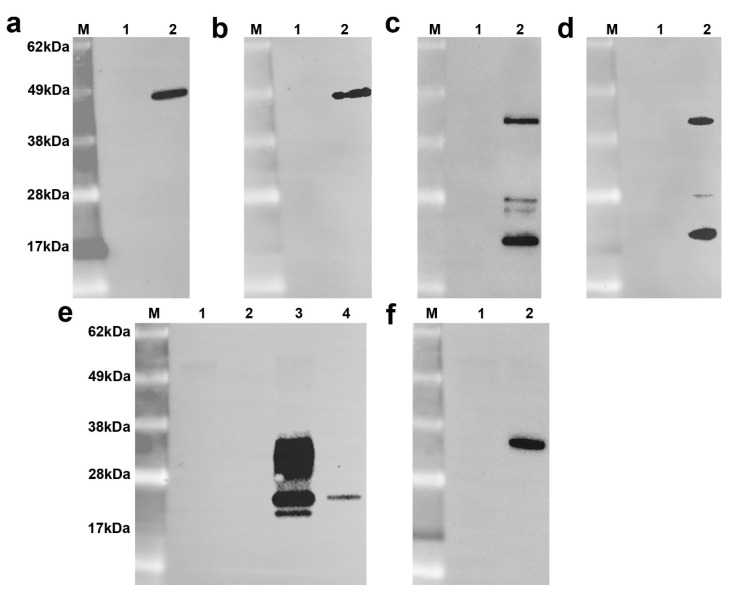
Immunoblot of BTV-1-infected cells using BTV antibodies specific for NS1, NS2, NS3/3a, and VP7. The blots shown were stained with NS1 monoclonal antibody (mAb) 31D11 (**a**), NS1 mAb 20A8 (**b**), NS2 mAb 30G4 (**c**), NS2 mAb 465 (**d**), NS3 mAb 441 (**e**), and VP7 polyclonal antibody 20-3 (**f**). The lanes for the images of NS1, NS2, and VP7 are as follows: molecular weight marker (M), mock-infected cell lysate diluted 1:8 (lane 1), and BTV-infected lysate diluted 1:8 (lane 2); and for NS3/3a blot, as follows: molecular weight markers (M), mock-infected cell lysate undiluted (lane 1) and diluted 1:8 (lane 2), and BTV-infected cell lysate undiluted (lane 3) and diluted 1:8 (lane 4).

Immunoblotting analyses showed minimal non-specific binding of the antibodies to mock infected cell lysate (Figure 3). Immunoblotting of BTV-1-infected cell lysates showed single protein bands for each of the NS1 and VP7 antibodies, with approximate molecular weights of 49 kDa (Figure 3a,b) and 36 kDa (Figure 3f), respectively, corresponding to the predicted sizes of the BTV NS1 and VP7 [38,39]. The monomeric NS2 band was identified at 46 kDa (Figure 3c,d) [40]. Lower molecular weight bands were also present, When the BTV-infected cell lysate (used undiluted, or diluted 1:8) was probed with NS3/3a-specific antibodies, a diffuse band of between 28 and 34 kDa corresponding to glycosylated NS3 was detected on the immunoblot above the bands corresponding to the non-glycosylated NS3 and NS3a (25 kDa and 24 kDa, respectively) (Figure 3e) [41]. Only a faint band corresponding to non-glycosylated NS3 (25 kDa) was detected using diluted BTV-infected cell lysate.

### 3.2. Immunoreactivity of BTV Antibodies against Diverse BTV Strains and Other Orbiviruses

**Figure 4:**
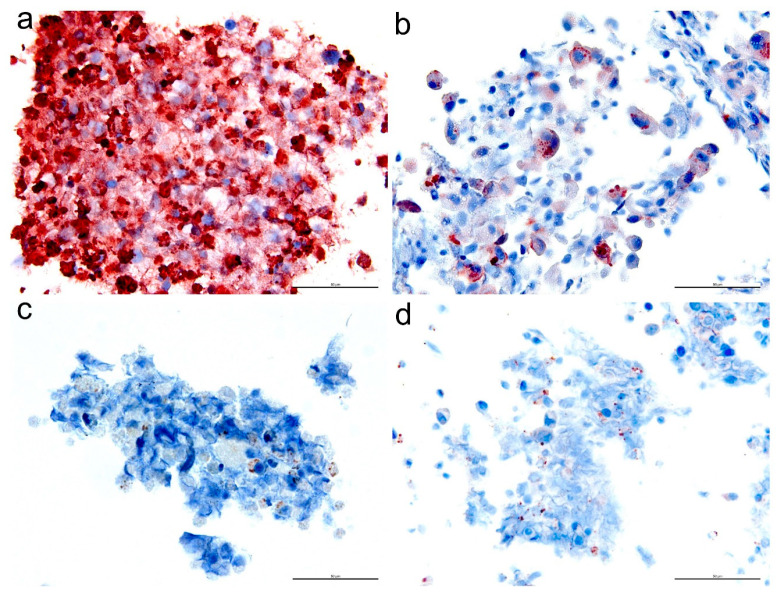
Immunolabelling of BHK-BSR cells infected with other orbiviruses using BTV NS2-specific mAb 465. Epizootic haemorrhagic disease virus (EHDV) (**a**), Eubenangee (**b**), Wallal (**c**), and Warrego virus (**d**). Bar = 50 μm.

We next sought to evaluate the immunoreactivity of selected antibodies to FFPE BHK-BSR cells infected with BTV and related orbivirus isolates belonging to a range of serotypes and from diverse geographical origins. To obtain diverse BTV isolates, we used our panel of available reference viruses, including isolates from South Africa, Australia, the Netherlands, USA, and Indonesia, representing both Eastern and Western topotypes (Table 2 and Table S1). The antibodies ultimately selected for characterization were as follows: 31D11, 20A8 (NS1), 30G4, 465 (NS2), 441 (NS3/3a), and 20-3 (VP7), as well as 53414-1 (NS2), which was previously tested for IHC [24]. The antibodies raised against NS1, NS2, and NS3/3a reacted with all BTV strains (Table 2). Bluetongue VP7 anti-sera detected all BTV strains except for those belonging to BTV-15. Immuno-detection using NS1 anti-sera did not produce labelling of the other orbiviruses tested. The BTV NS2 mAb 465 (Figure 4a) and NS3/3a mAb 441 produced strong immunolabelling on EHDV-infected cells, whereas weak immunolabelling was observed for the polyclonal NS2 and VP7 antibodies. The BTV NS2 mAb 465 (Figure 4b), NS3/3a mAb 441, and VP7 pAb 20-3 were also weakly cross-reactive to Eubenangee virus-infected cells (Table 2). Furthermore, sparse immunolabelling was observed in cells infected with Wallal and Warrego viruses that were stained with BTV NS2 polyclonal antibody 53414-1, mAb 465 (Figure 4c,d), NS3/3a mAb 441, and VP7 polyclonal antibody.

## 4. Discussion

This report describes the application of immunohistochemistry to FFPE BTV-infected cells and sheep lung for the reliable detection of viruses within the BTV serogroup. Formalin is routinely used in histology laboratories for the fixation of tissues due to its reliable properties for the inactivation of infectious agents in tissues and excellent preservation of tissue morphology. However, cross-linking of antigens caused by formaldehyde fixation can mask immunogenic epitopes, thus hampering or eliminating antibody binding [42]. Earlier attempts with immunolabelling of BTV in tissues produced variable outcomes on FFPE sections [43]. In our protocol, as well as those described by other investigators, heat-mediated antigen retrieval was used to re-expose the epitopes [24,25,26]. As the detection of BTV antigens by IHC can suffer from low sensitivity [43], polymer-based visualization involving numerous HRP molecules conjugated to the polymer backbone was also adopted in this study to enhance the detection [42]. Using this methodology, the detection of BTV antigens can be sensitively and reliably reproduced by a range of BTV antibodies.

In order to investigate the potential for broader application of BTV antibodies, the immunoreactivity of antibodies raised against BTV NS1, NS2, NS3/NS3a, VP2, and VP7 was evaluated for the detection of orbiviruses belonging to different serotypes and from diverse geographic origins. The majority of the VP2 antibodies tested in this study were previously used for serological assays [33]. Among the collection of VP2 antibodies, only mAb 30E3/F4 has been successful described for application to immunoelectron microscopy (IEM) and immunofluorescence (IF) for detecting virus particles [35]. However, in our hands, no VP2 antibodies were successful in detecting antigen in FFPE sections. Although other antigen retrieval conditions such as heat-mediated pH6 or enzymatic-mediated retrieval methods were not attempted for the detection of VP2 by IHC [42], the inability of VP2 antibodies to recognise various immunogenic sites, as described previously for neutralisation, IEM, or IF, suggests that VP2 epitopes were masked by formalin fixation and paraffin embedding, and thus were not suitable for IHC detection on FFPE specimens.

The NS1 mAbs demonstrated BTV serogroup specificity and no cross-reactivity was detected against other orbiviruses (Table 2), consistent with earlier studies reporting that BTV NS1 antisera were not immunoreactive against EHDV and AHSV [44,45]. The linear epitopes of NS1 harbor conserved residues among the immuno-reactive epitopes within the BTV species, but these are not shared with EHDV and AHSV [46]. Previously, the in-house produced NS1 mAbs 31D11 and 20A8 were utilized in IEM studies, in which these mAbs were reported to demonstrate labelling of virus specific tubules [36].

Several antibodies against BTV NS2 and NS3/NS3a were reliably immunoreactive against BTV. However, they were also immunoreactive against other related orbiviruses, albeit with weaker labelling, suggestive of partial conservation of the relevant immunoreactive epitopes. Besides the conserved nature of the NS2 protein within the BTV serogroup, significant amino acid conservation exists within viruses belonging to the *Orbivirus* genus, especially at the N-terminal domain where BTV has the closest antigenic relationship with EHDV [19]. An earlier study revealed high amino acid similarity between BTV and EHDV at the N-terminus, where the amino acid positions 1 to 97 and 117 to 166 had 79% and 84% identity, respectively [47]. Subsequent studies also confirmed that the NS2 protein is highly conserved among BTV and other related orbiviruses such as EHDV, AHSV, PHSV, Chuzan virus, and Yunnan orbivirus-2 at the N-terminal domain [19]. In our investigation, the NS2-specific mAb 465 reacted strongly to EHDV, but weakly to other orbiviruses in formalin-fixed cells, whereas the NS2 mAb 30G4 did not react to other orbiviruses apart from BTV. This suggested the two NS2 mAbs used in this study bind to different epitopes. Low molecular weight bands of BTV-infected cell lysates were detected by immunoblotting using the two NS2 mAbs (Figure 3c,d). These bands likely represented breakdown products as protease inhibitors were not included in the sample preparation process. The absence of low molecular weight bands in immunoblots of uninfected cell lysates and the lack of NS2 immunolabelling on FFPE sections of uninfected cell pellet, together with the different patterns of immunoreactivity (Table 2), indicated specific binding of these mAbs to the low molecular weight bands.

Bluetongue virus NS3/3a proteins are also highly conserved across BTV serotypes, with significant residue conservation with other orbiviruses at both the N- and C-termini [20]. Despite the conservation of NS3 peptide among orbiviruses, BTV and EHDV share higher levels of amino acid similarity than that between BTV and AHSV [48]. In one study, a NS3/3a mAbs that recognized the region between amino acid positions 82 to 95 of BTV NS3/3a cross-reacted with 24 serotypes of BTV as well as Ibaraki virus (a strain of EHDV) [20]. The cross-reactivity of the NS3/3a mAb 441 with EHDV and other orbiviruses tested in our study is consistent with this earlier report and reflects the conserved nature of the NS3/3a epitope.

The BTV genome segment 7 that encodes for VP7 determines the defining epitopes for the serogrouping of BTV and other orbivirus species [14]. A BTV serogroup specific mAb, clone 20E9/B7/G2, previously shown to detect core protein VP7 on IEM [37], was unsuccessful in the current application of IHC on FFPE BTV infected cells. In our study, of the VP7 antibodies tested, only the rabbit polyclonal antibody raised against BTV-1 VP7, 20-3, was able to detect BTV in FFPE specimens. In addition to BTV, this antiserum also demonstrated the ability to detect other orbiviruses such as EHDV in an IHC format. It is known that certain immunogenic epitopes of BTV and EHDV are highly conserved and cross-reactivity occurs with BTV VP7 mAbs [49]. Although VP7 is highly conserved within the BTV species, with up to 90% amino acid identity, significant variation can still be found [50]. The rabbit polyclonal antibody used in our study did not cross-react with either the Australian or the South African reference BTV-15 isolates (Table 2), which is consistent with an earlier finding that mAbs raised against BTV-1 and BTV-16 failed to detect BTV-15 [51]. BTV-15 possesses an additional cysteine residue at position 105 and two lysine residues at positions 58 and 324 that are likely to impart significant changes to protein conformation [52]. Therefore, homotypic antisera will be needed for the detection of BTV-15 VP7.

Our extensive evaluation of selected BTV antibodies has demonstrated their versatility to detect other orbiviruses. This property could be beneficial for generic orbivirus detection, but may also produce equivocal results if a test subject was co-infected with more than one species of *orbivirus*. In using these antibodies for IHC, diagnosticians always need to consider that such cross-reactions are possible and incorporate that into their interpretation. The use of IHC described in this study should be evaluated for fitness-for-purpose prior to the application in research or diagnostic settings. To comprehensively characterise the extent of antibody reactivity, we endeavoured to maximize orbivirus species diversity. Thus, we accessed all isolates of Western origin BTV topotypes currently available at our laboratory as well as a full complement of prototype Australian BTV (Eastern topotype) and other available orbiviruses. We were also cognizant of the likelihood of diversity within each of the proteins occurring over time, through antigenic drift during geographic isolation and antigenic shift through genetic reassortment. Although it may have been useful to determine variation, protein sequence characterisation of each antigens of interest was beyond the scope of the study.

In conclusion, antibodies generated against BTV non-structural and structural proteins were able to detect BTV antigens in infected cells and sheep lung that were formalin-fixed and paraffin-embedded. We have shown that the NS2 and NS3/3a are the preferred target antigens for detection of BTV by IHC on FFPE material. Antibody detection of NS1 and VP7 was, however, less sensitive. The detection of VP2 under these conditions is not recommended as none of the antibodies tested were shown to be reactive. The largely conservative nature of selected BTV antigens also indicated that numerous BTV strains prepared in cell pellets as described in our methods can be used as positive control material for the development of IHC assay. Importantly, our work has also showed that FFPE infected cell pellets can be successfully used as a substitute for infected animal tissues to determine optimal antigen targets and to characterise antibodies for IHC.

## Figures and Tables

**Table 1:** List of antibodies screened for immunoreactivity to bluetongue virus (BTV)-1-infected formalin-fixed paraffin-embedded cell pellet by immunohistochemistry.

Antibody (Abbreviation)	Isotype	Previous Application [Reference]	BTV Antigen	Clone	Immunoreactivity	Origin
31D8/A12/D9	IgG	ELISA, VNT [33]	VP2	mAb *	-	ACDP ^#^
31C10/C4	IgG3	n/a			-	
31D8/A12/D2	n.d.	ELISA, VNT [33]			n.s	
30E6/G4	IgG2a	ELISA, VNT [33,34]			-	
31D8(6)	n.d.	n/a			-	
30E3/F4	IgG2b	ELISA, VNT, IEM, IF [34,35]			-	
31A2/D2	IgG3	ELISA, VNT [33]			-	
31D8/A12/H3	n.d.	ELISA, VNT [33]			n.s	
20A11/10	IgG1	ELISA, IEM, IF [34,36]	VP7	mAb	-	
20D9/F12	IgG2a	n/a			-	
20E9/B7/G2	IgG2a	ELISA, IEM, IF [34,37]			-	
PKA4	IgGa	n/a			-	
20-3	IgG	n/a		pAb ^	+	
31D11/B10 (31D11)	IgG3	IEM [36]	NS1	mAb	+	
20A810 (20A8)	IgG2a	ELISA, IEM [34]			+	
30G4/B10 (30G4)	IgG2a	ELISA [34]	NS2	mAb	+	
465	IgG	IF [32]			+	LSHTM †
53414-1	IgG	IHC, IF [24]		pAb	+	Glasgow
441	IgG	IF [32]	NS3	mAb	+	LSHTM

+ positive; - negative; n.s. = non-specific; * mAb = mouse monoclonal antibody; ^ pAb = rabbit polyclonal antibody; n.d. = not determined; ELISA = enzyme-linked immunosorbent assay; IEM = immunoelectron microscopy; VNT = virus neutralisation test; IF = immunofluorescence; IHC = immunohistochemistry; ^#^ CSIRO Australian Centre for Disease Preparedness (ACDP); † London School of Hygiene and Tropical Medicine (LSHTM); University of Glasgow (Glasgow).

**Table 2:** Histochemical immunoreactivity of infected baby hamster kidney cells (BHK-BSR) using antibodies against a range of BTV isolates and other orbivirus species from diverse geographic origins.

Virus		Antibodies						
		NS1		NS2			NS3/3a	VP7
Origin	Species	31D11 mAb	20A8 mAb	53414 -1 pAb	465 mAb	30G4 mAb	441 mAb	20-3 pAb
**South Africa ^**	BTV	+	+	+	+	+	+	+/- ^1^
**USA**	BTV	+	+	+	+	+	+	+
Australia *	BTV	+	+	+	+	+	+	+/- ^1^
**Netherlands**	BTV	+	+	+	+	+	+	+
Australia *	EHDV ^a^	-	-	(+)	+	-	+	(+)
	Wallal	-	-	(+)	(+)	-	(+)	(+)
	Warrego	-	-	(+)	(+)	-	(+)	(+)
	Eubenangee	-	-	-	(+)	-	(+)	(+)
	YUOV-2 ^b^	-	-	-	-	-	-	-
	PHSV ^c^	-	-	-	-	-	-	-
**Indonesia**	Palyam	-	-	-	-	-	-	-
**South Africa**	AHSV ^d^	-	-	-	-	-	-	-

+ positive; (+) weak positive; - negative; mAb = monoclonal antibody; pAb = polyclonal antibody; ^ reference strains; * prototype strains; ^1^ negative immunoreactivity for BTV-15; ^a^ epizootic haemorrhagic disease virus (EHDV); ^b^ Yunnan orbivirus 2 (YUOV-2); ^d^ African horse sickness virus (AHSV); ^c^ Peruvian horse sickness virus (PHSV).

## Supplementary Materials

Supplementary materials can be found at https://www.mdpi.com/2076-2607/8/8/1207/s1. Table S1. Orbivirus isolates used for the generation of FFPE infected cell pellets.
