# Supplementary material for: Evaluation of Bluetongue Virus (BTV) Antibodies for the Immunohistochemical Detection of BTV and Other Orbiviruses
Source: Microorganisms. 2020 Aug 7;8(8):1207. doi: 10.3390/microorganisms8081207 (PMC7464351; doi:10.3390/microorganisms8081207)
Supplement: Supplementary file 1 [file microorganisms-08-01207-s001.pdf]

Table 1. Orbivirus isolates used for the generation of FFPE infected cell pellets.

Origin	Orbivirus Species (Isolate)
Reference Strains (South Africa)	BTV1 (RSArrrr/01)
	BTV2 (RSArrrr/02)
	BTV3 (RSArrrr/03)
	BTV4 (RSArrrr/04)
	BTV5 (RSArrrr/05)
	BTV6 (RSArrrr/06)
	BTV7 (RSArrrr/07)
	BTV8 (RSArrrr/08)
	BTV10 (RSArrrr/10)
	BTV11 (RSArrrr/11)
	BTV12 (RSArrrr/12)
	BTV13 (RSArrrr/13)
	BTV14 (RSArrrr/14)
	BTV15 (RSArrrr/15)
	BTV16 (RSArrrr/16)
	BTV18 (RSArrrr/18)
	BTV19 (RSArrrr/19)
	BTV22 (RSArrrr/22)
	BTV24 (RSArrrr/24)
USA	BTV2
	BTV10
	BTV11
	BTV17
Netherlands	BTV8 (Net2006-A)
Australia (Prototype Strains)	BTV1 (CSIRO156)
	BTV2 (DPP7291)
	BTV3 (DPP973)
	BTV5 (V9230)
	BTV7 (DPP6963)
	BTV9 (DPP836)
	BTV12 (V9244)
	BTV15 (DPP192)
	BTV16 (P16-02109-0009)
	BTV20 (CSIRO19)
	BTV21 (CSIRO154)
	BTV23 (DPP90)
	EHDV1 (V2209)
	Wallal virus (VIAS)
	Warrego virus (AA001/95)
	Eubenangee virus (CS32)
	Yunnan orbivirus-2 [Middle point] (V4353)
	Peruvian horse sickness virus PHSV [Elsey] (99/724a)
Indonesia	Palyam (RIVS130)
South Africa	AHSV2
	AHSV4
